# **Correlation between spatial (3D) structure of pea and bean thylakoid membranes and arrangement of chlorophyll-protein complexes**

**DOI:** 10.1186/1471-2229-12-72

**Published:** 2012-05-25

**Authors:** Izabela Rumak, Radosław Mazur, Katarzyna Gieczewska, Joanna Kozioł-Lipińska, Borys Kierdaszuk, Wojtek P Michalski, Brian J Shiell, Jan Henk Venema, Wim J Vredenberg, Agnieszka Mostowska, Maciej Garstka

**Affiliations:** 1 Department of Plant Anatomy and Cytology, Institute of Plant Experimental Biology and Biotechnology, Faculty of Biology, University of Warsaw, Miecznikowa 1, Warsaw, PL-02-096, Poland; 2 Department of Metabolic Regulation, Institute of Biochemistry, Faculty of Biology, University of Warsaw, Miecznikowa 1, Warsaw, PL-02-096, Poland; 3 Department of Biophysics, Institute of Experimental Physics, Faculty of Physics, University of Warsaw, Żwirki i Wigury 93, Warsaw, PL-02-089, Poland; 4 Australian Animal Health Laboratory, CSIRO Livestock Industries, 5 Portarlington Road Geelong, Victoria, 3220, Australia; 5 Laboratory of Plant Physiology, Centre for Ecological and Evolutionary Studies (CEES), University of Groningen, P.O. Box 11103, Groningen, 9700 CC, The Netherlands; 6 Department of Plant Physiology, Wageningen University and Research Centre, Wageningen, 6708 PB, The Netherlands

## Abstract

**Background:**

The thylakoid system in plant chloroplasts is organized into two distinct domains: grana arranged in stacks of appressed membranes and non-appressed membranes consisting of stroma thylakoids and margins of granal stacks. It is argued that the reason for the development of appressed membranes in plants is that their photosynthetic apparatus need to cope with and survive ever-changing environmental conditions. It is not known however, why different plant species have different arrangements of grana within their chloroplasts. It is important to elucidate whether a different arrangement and distribution of appressed and non-appressed thylakoids in chloroplasts are linked with different qualitative and/or quantitative organization of chlorophyll-protein (CP) complexes in the thylakoid membranes and whether this arrangement influences the photosynthetic efficiency.

**Results:**

Our results from TEM and *in situ* CLSM strongly indicate the existence of different arrangements of pea and bean thylakoid membranes. In pea, larger appressed thylakoids are regularly arranged within chloroplasts as uniformly distributed red fluorescent bodies, while irregular appressed thylakoid membranes within bean chloroplasts correspond to smaller and less distinguished fluorescent areas in CLSM images. 3D models of pea chloroplasts show a distinct spatial separation of stacked thylakoids from stromal spaces whereas spatial division of stroma and thylakoid areas in bean chloroplasts are more complex. Structural differences influenced the PSII photochemistry, however without significant changes in photosynthetic efficiency. Qualitative and quantitative analysis of chlorophyll-protein complexes as well as spectroscopic investigations indicated a similar proportion between PSI and PSII core complexes in pea and bean thylakoids, but higher abundance of LHCII antenna in pea ones. Furthermore, distinct differences in size and arrangements of LHCII-PSII and LHCI-PSI supercomplexes between species are suggested.

**Conclusions:**

Based on proteomic and spectroscopic investigations we postulate that the differences in the chloroplast structure between the analyzed species are a consequence of quantitative proportions between the individual CP complexes and its arrangement inside membranes. Such a structure of membranes induced the formation of large stacked domains in pea, or smaller heterogeneous regions in bean thylakoids. Presented 3D models of chloroplasts showed that stacked areas are noticeably irregular with variable thickness, merging with each other and not always parallel to each other.

## Background

The thylakoid system in plants is organized into two distinct domains: grana arranged in stacks of appressed membranes and non-appressed membranes consisting of stroma thylakoids and margins of granal stacks [1]. It is known that appressed membranes that form grana are not essential for photosynthesis but they are ubiquitous in all chlorophyll (Chl) *b*-containing higher plants [1,2]. Many photosynthetic organisms such as red algae, Cyanobacteria, many green algae such as *Chlamydomonas reinhardtii* have no stacked thylakoids. Apart from higher plants only Charophyta have appressed membranes indistinguishable from those of land plants [1].

Why did plants develop grana? The development of appressed membranes caused structural heterogeneity that is reflected by functional differentiation with respect to the location of hierarchically organized photosyntetic complexes in supercomplexes and megacomplexes within appressed and non-appressed membranes [3,4]. Size and charge differences between PSI and PSII play a key role in their lateral arrangement [5,6]. Photosynthetic unit PSIIα, i.e., LHCII-PSII supercomplex occurs exclusively in appressed regions and is composed of the dimer of the PSII core, minor light-harvesting complexes (Lhcb4-6) and variable amounts of LHCII trimers (Lhcb1-3) [4,7-9]. In non-appressed thylakoid regions the monomeric PSI core complex with four LHCI subunits (Lhca1-4) and with temporarily bound LHCII complex form LHCI-PSI supercomplexes [7,10]. In addition the photosynthetic unit PSIIβ, i.e. monomeric PSII without LHCII trimers, exists in stroma thylakoids [4,7]. The structural and organizational changes of grana stacks are driven by physical and chemical forces. It is believed that membrane appression is maintained primarily by the balance between the van der Waals attraction versus electrostatic and hydratation repulsion [6].

It is argued that the reason for the development of appressed membranes in plants is that their photosynthetic apparatus needs to cope with and survive ever-changing environmental conditions, such as transition from darkness, low-light to high-light conditions [1] or temperature fluctuation [11,12]. Short-term changes are due to the redistribution of absorbed excitation energy (state to state transition) that is based on migration of LHCII from PSII after its phosphorylation [13-15]. Tightly appressed arrangement of thylakoid membranes results in high stability of the chloroplast structure, which needs to be somehow combined with high flexibility/adaptability to dynamically changing environmental conditions [6].

Why do different plant species have different arrangements of grana within their chloroplasts? Examinations of mutants yielded some information on the arrangement of thylakoid membranes within chloroplasts. For example it is known that Arabidopsis *Aba* mutants – deficient in epoxy-carotenoids – have significantly more grana stacks per chloroplast and more chloroplasts per cell but reduced thylakoid stacking in comparison with wild Arabidopsis plants [16]. In tobacco knockdown of PsbP protein, one of the three oxygen evolving complex proteins in plants, impairs the accumulation of PSII supercomplexes in tobacco and causes large disorder in the thylakoid grana stacking [17]. Studies of the arrangements of thylakoid membranes gave information on chlorophyll *b* –less Arabidopsis mutants. Markedly decreased level or the absence of most of Lhcbs caused fewer grana and much longer stromal thylakoids than in the wild type which implied that the total granal cross-sectional area per chloroplast area was decreased dramatically [18]. Ruban and coworkers [19] argued that in the absence of one of the PSII supercomplex main components, another antenna protein may be recruited to replace a mutated or absent protein, therefore allowing the main complex to assemble and function correctly.

Mutations in the photosynthetic apparatus that cause changes in the thylakoid arrangement give an altered picture of chloroplast thylakoid membranes with profound effect on the photosynthetic efficiency and capacity [18]. Data on the thylakoid arrangement in chloroplasts of different plant species grown in natural or varying environmental conditions, can supplement information obtained for the mutants. Thus answering a question whether and how structural heterogeneity at different levels of thylakoid organization can relate to adaptation/acclimation to changing environment and better photosynthetic efficiency is of significant importance.

In this paper we propose possible interpretation at molecular level of different arrangements of the thylakoid membranes of pea (*Pisum sativum* L.) and bean (*Phaseolus vulgaris* L.) grown under similar controlled conditions, as a continuation of our previous studies [11,12]. Here we compared different chloroplast structures by confocal laser scanning microscopy (CLSM) followed by computer modeling and by transmission electron microscopy (TEM). The arrangement and composition of chlorophyll-protein (CP) complexes were examined by mild-denaturing electrophoresis, SDS-PAGE, tandem mass spectrometry (MS/MS), immunodetection and by low-temperature steady-state spectrophotometry. Additionally PSII activity was analyzed by modulated and transient Chl *a* fluorescence.

By applying these diverse methodologies we attempted to describe the relationship between spatial chloroplast structure detected by CLSM *in situ* and the arrangements of CP complexes within the thylakoid membranes. Our aim was to determine at the molecular level, whether different arrangement and distribution of appressed and non-appressed thylakoids in chloroplasts are linked with different qualitative and/or quantitative organization of CP complexes in the thylakoid membranes and whether it influences the photosynthetic efficiency.

## Results

### CLSM images and three dimensional (3D) reconstruction of chloroplast structure

**Figure 1:**
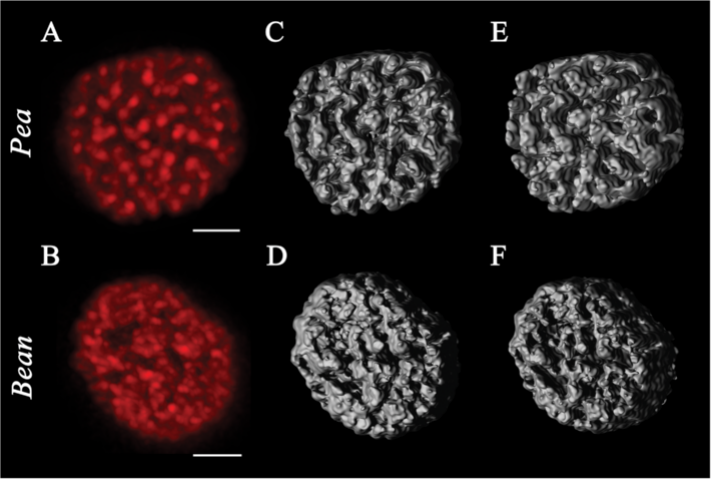
**Chlorophyll fluorescence of intact chloroplasts revealed by CLSM and 3D reconstruction of chloroplast structure.** The intact chloroplasts of pea (**A**) and bean (**B**) were incubated in isoosmotic medium containing 15 mM NaCl and 4 mM MgCl_2_. Each red image presents the maximum intensity projection of deconvolved stack of CLSM images. Bar = 2 μm. Images are representative for at least 20 independent experiments. Grey images represent 3D models of intact pea (**C, E**) and bean chloroplasts (**D, F**) created after deconvolution. Face (**C, D**) and side view (**E, F**) of 3D chloroplast models is shown. Each image is a representative of at least 10 independent experiments.

CLSM images revealed fluorescent red spots of about 0.5 μm separated from one another by dark spaces inside pea chloroplasts (Figure 1A). As it was demonstrated by others [20-22] these bright spots reflected mainly appressed thylakoid membranes containing LHCII-PSII supercomplexes and LHCII trimers rather than non-appressed thylakoids containing PSI. In bean chloroplasts (Figure 1B) smaller and less distinguished fluorescent discs were observed (Figure 1A, B).

Large number (94–116) of fluorescence images were taken in different focal depths. This allowed creating computer-generated 3D structures in which the spatial layout of Chl fluorescence was shown (Figure 1C – F). In pea chloroplasts, the spatial layout of Chl fluorescence consisted of distinguished sharply falling down edges forming deep, curved gorges corresponding to the dark areas in CLSM images (Figure 1C, E). The surface of Chl fluorescence areas is seen in the face view as well as in the side view. 3D images of bean chloroplasts were less regular, with edges not as sharp as in pea, resulting in shallow, irregular caves corresponding to the dark, non-fluorescent areas between the red bodies observed by CLSM (Figure 1D, F). In each 3D structure one can find a corresponding detail of the CLSM image. For a better view of chloroplast 3D structures, animated models are included in Additional file 1: Video S1 and Additional file 2: Video S2. The 3D structures reflect the distribution of Chl fluorescence in a chloroplast as a whole.

### TEM chloroplast structure in mesophyll cells

**Figure 2:**
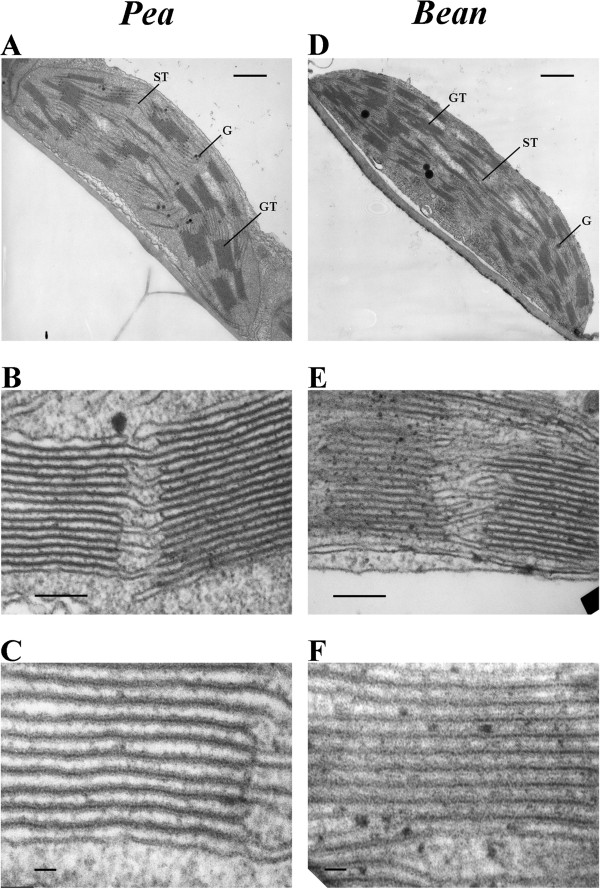
**Mesophyll chloroplast images revealed by TEM.** Pictures show different thylakoid arrangements in pea (**A**) and bean chloroplasts (**D**), larger appressed thylakoids regions in pea (**B**) than in bean (**E**) and wider thylakoid lumen in pea (**C**) than in bean (**F**). Bar = 500 nm (**A, D**); 100 nm (**B, E**); 20 nm (**C, F**).

Images of pea chloroplasts of leaf mesophyll from TEM showed large appressed thylakoid regions (grana), clearly separated from one another by non-appressed ones and oriented paralelly to one another (Figure 2A). These large appressed thylakoids in pea chloroplasts (Figure 2B) correspond to large, uniformly distributed red fluorescent 0.5 μm bodies seen by CLSM (cf. Figure 1A). Bean chloroplasts contain numerous non-appressed thylakoid regions with some appressed ones which are smaller than those in pea thylakoids and are irregularly distributed within chloroplasts (Figure 2D, E). These appressed regions in TEM images of bean chloroplasts are counterparts of smaller and less distinguished fluorescent areas seen by CLSM (cf. Figure 1B).

The average value of the ratio of the length of grana thylakoids to the length of stroma thylakoids was 3.73 ± 0.24 in pea while in bean it was only 2.77 ± 0.18. The average height ratio of a granum to the number of thylakoids in a particular granum was also bigger in pea (Figure 2B) than in bean chloroplasts (Figure 2E). The distance between the pairs of membranes in thylakoids was estimated to be 20.45 ± 0.68 nm in pea and 15.63 ± 1.57 nm in bean chloroplasts. This reflected a relatively larger thylakoid lumen in pea thylakoids (Figure 2C) compared with bean thylakoids (Figure 2F). The chloroplast images from intact chloroplasts by CLSM and from leaf tissue *in situ* by TEM were consistent with each other and gave similar information regarding different distribution and appearance of appressed and non-appressed thylakoids in pea and bean chloroplasts.

### Protein composition

**Figure 3:**
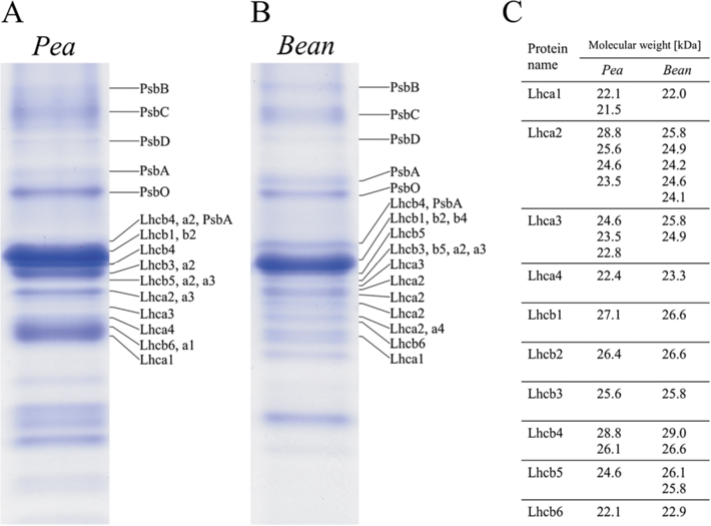
**Protein analysis of pea and bean thylakoid membranes.** SDS-PAGE resolution of thylakoid membrane proteins (9 μg of chlorophyll) and visualized by staining with Coomassie Blue R-250 (**A, B**). Separated bands were analyzed by immunodetection and identified by mass spectrometry. Molecular weights [kD] of pea and bean Lhca1-4 and Lhcb1-6 are given in the table (**C**).

Separation of thylakoid membrane components by SDS-PAGE in linear gradient gels (14-20 %) resulted in a very high resolution of individual proteins from both pea and bean. More than 50 proteins from 10 to 180 kD in molecular weight were distinguishable (Additional file 3: Figure S1). The thylakoid membrane proteins in 15–45 kD molecular weight range are presented in Figure 3. Following electrophoretic separation the proteins of interest were identified by immunodetection and MS/MS analysis.

Electrophoretic pattern was similar in pea and bean thylakoid samples and proteins PsbA, PsbB, PsbC, PsbD, PsbO, Lhca1-4 and Lhcb1-6 were identified in both samples (Figure 3A, B). However significant compositional differences were observed in the gel area where the antennae proteins (Lhca and Lhcb) were localized (20–30 kD). Ten bands in pea (Figure 3A) and 12 bands in bean samples (Figure 3B) were distinguished in this region. Moreover, pea and bean Lhca1, Lhca2, Lhca3, Lhcb4 and Lhcb5 polypeptides were found in several bands (Figure 3A, B). The main qualitative differences between the pea and bean antennae proteins concern a number of isoforms of Lhca1-3 and their molecular weights (Figure 3C).

**Figure 4:**
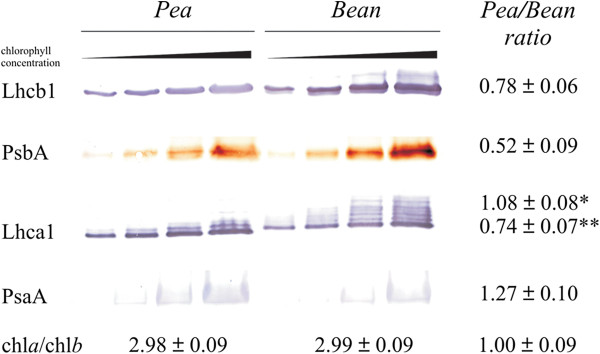
**Quantitative immunodetection analysis of PSII and PSI antenna (Lhcb1, Lhca1) and core (PsbA, PsaA) proteins.** Pea and bean thylakoid samples of increasing amount of chlorophyll starting from 0.25 or 0.5 to 2 or 4 μg for antenna and core proteins, respectively were separated by SDS-PAGE. Pea/bean ratios are calculated on the basis of pixel intensity analysis and are mean values ± SD for three to five separate experiments. The presented Chl *a*/Chl *b* ratios are mean value ± SD for at least fifteen independent measurements. Pea/bean ratio for Lhca1 protein was determined for main (*) or all (**) detected bands.

The relative amounts of PSII and PSI antennae and the core proteins compared to the total Chl (*a* + *b*) amount were determined by immunodetection with specific antibodies (Figure 4). For more accurate estimation each sample was examined at four different chlorophyll concentrations (Figure 4). Quantitative relationships between the two samples were expressed as a ratio of pixel intensities corresponding to selected proteins bands normalized to chlorophyll content. Lhcb1 was detected as one major band in pea and one major and two minor bands in bean samples. The presence of these two additional bands in bean sample is probably an effect of anti- Lhcb1 antibody cross interactivity with the Lhcb4 (top band) and the Lhcb3 (bottom band) proteins (cf. Figure 3B). Because of apparent low antibody specificity against bean Lhcb1, quantitative analysis was performed only for the major band which was about 30 % more abundant in bean than in pea. On the other hand the level of PsbA in bean was about two times higher than in pea, which suggests a higher ratio of Lhcb1 to PsbA in pea thylakoids. The Lhca1 detection pattern consisted of one dominant and three (pea) or six (bean) minor bands with higher molecular weight. These additional bands may correspond to different isoforms of Lhca1 [23], (Figure 3). There was no difference between the levels of Lhca1 main band in pea and bean samples but pea/bean ratio for all detected Lhca1 bands indicated lower contents in pea, which can be explained by a difference in detected isoforms. PsaA, the PSI core protein, was detected as 55–60 kD band whereas expected molecular weight for this protein is around 83 kD [24]. The PsaA level in pea thylakoids is similar to bean up to the standard deviation.

The Chl *a*/Chl *b* ratios were the same in pea and bean thylakoids (Figure 4), hence the detected differences in antenna/core protein ratios are likely to be related to protein proportion within the thylakoid membranes. Comparison of Lhca1 and PsaA ratios suggests that the amounts of LHCI-PSI were similar in the thylakoids of both species. Due to statistically significant difference in Lhcb1 and PsbA ratios we can explicitly conclude that the proportion of PSII antennae to the core proteins was higher in pea than in bean thylakoids. However unambiguous estimation of PSII-LHCII abundance was more complicated because: (i) part of LHCII might be arranged in an aggregated form, (ii) from two up to six LHCII trimers might be associated with a PSII dimer and (iii) PsbA protein is partially localized in PSII monomers which are not connected to LHCII antennae [4,7]. Therefore the mild-denaturing electrophoresis and 77 K fluorescence spectra were performed for detail analysis.

### The chlorophyll-protein complexes composition

**Figure 5:**
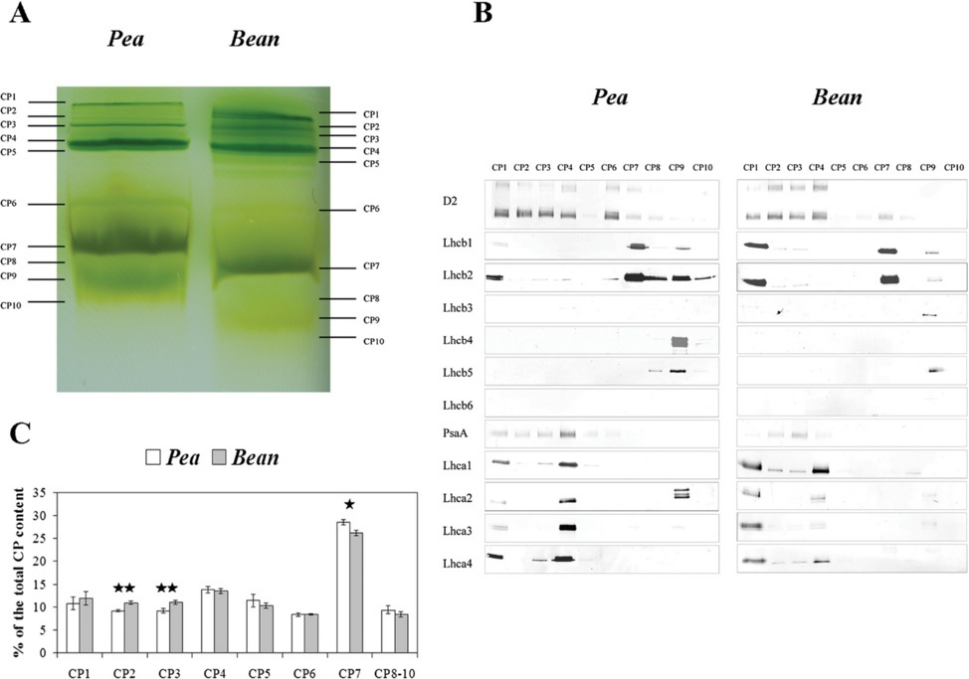
**Mild-denaturing “green” gel electrophoresis of CP complexes isolated from pea and bean leaves.** The presented “green” gels are representative for at least seven independent experiments (**A**). Immunodetection of proteins that are the part of CP bands were resolved by mild-denaturing electrophoresis (**B**). Relative intensities of CP bands resolved by mild-denaturing electrophoresis, quantified by Quantity One software (**C**) The percentage amounts of CP complexes are mean values ± SD for 3 independent experiments. *P < 0.01, **P < 0.05.

The chlorophyll-protein complexes were gently released from the membranes by n-decyl-β-D-maltopyranoside and n-octyl-β-D-glucopyranoside, and separated by mild-denaturing ‘green’ gel electrophoresis. The separation of pea and bean thylakoids revealed 10 green bands assigned to ten different CP complexes (CP1-CP10) (Figure 5A). According to Allen and Staehelin [25], CP1-CP5 bands correspond to LHCI-PSI and LHCII-PSII supercomplexes. CP6 corresponds to reaction center complexes and includes PSI and PSII complexes without external antennae system attached. Band CP7 is assigned to LHCII supercomplexes. CP8-CP10 bands correspond to monomers (Figure 5A). The pea and bean complexes differed in the electrophoretic mobility of their components, especially of CP7 to CP10 that appeared to have higher molecular weights in pea thylakoids.

Immunodetection analysis of PsbD (also named D2), PsaA, Lhcb1-5 and Lhca1-4 proteins with specific antibodies was performed to study the polypeptide composition of the chlorophyll-protein complexes (Figure 5B). Generally all those proteins, except for Lhcb3, Lhcb4, Lhcb5, were found in pea and bean thylakoids in CP1 to CP4 bands (Figure 5A). It supports statement that the CP1-CP4 bands could contain LHCI-PSI and LHCII-PSII supercomplexes. Immunodetection of D2, PsaA, Lhca1, Lhca4 within pea CP5 band was low and even lower in bean CP5. In the CP6 band D2, PsaA and Lhcb2 were detected in pea but only D2 in bean. In CP7 mainly Lhcb1 and Lhcb2 were detected in pea and bean; and it is well known that these two proteins build functional LHCII supercomplexes. In this band a weak detection of D2, PsaA, Lhca3 in pea and D2 and Lhca1 in bean was observed. In the monomer region (CP8-CP10) many of individual proteins were resolved.

Quantitative analysis of mild-denaturing electrophoresis pattern showed significant differences in chlorophyll-protein complexes isolated from pea and bean leaves. The amount of CP2 and CP3 containing LHCI-PSI and LHCII-PSII supercomplexes was considerably lower in pea than in bean thylakoids (Figure 5C). Moreover, the proportion of CP7 assigned to LHCII complexes was higher in pea than in bean (Figure 5C). Because the mild-denaturing electrophoresis did not preserve completely the CP supercomplexes structure [25], the presented results illustrate the stability of distinct CP complexes after the detergent treatment rather than its native organization in thylakoid membranes. Thus, the intensity of CP7 bands (Figure 5A) is related to LHCII associated loosely or strongly with LHCII-PSII supercomplexes and also with LHCII which is not directly associated with PSII. Thus this indicates again that the intensities of CP2 and CP3 bands (Figure 5A) are a consequence of its stability, probably due to restricted detergent accessibility to the CP complexes.

### Analysis of the relative composition of the pea and bean thylakoid membranes by low-temperature spectrophotometry

**Figure 6:**
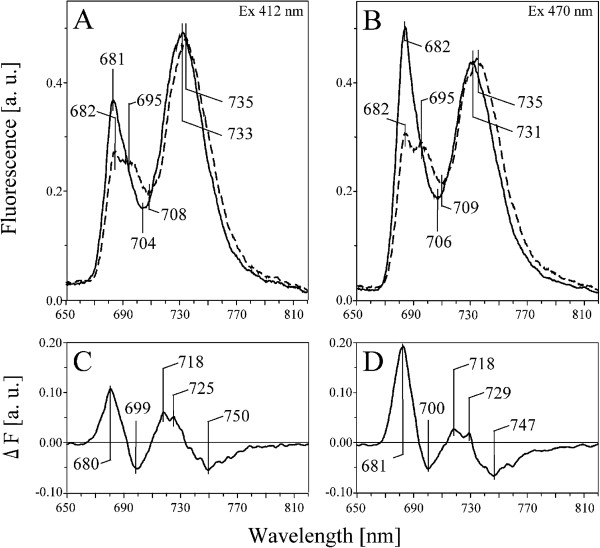
**Fluorescence emission spectra at 77 K of pea and bean thylakoids.** Emission spectra excited at 412 (**A**) and 470 nm (**B**) for pea (solid line) and bean (dashed line) thylakoids (10 μg Chl ml^-1^). Fluorescence emission-difference spectrum pea-minus-bean for samples excited at 412 (**C**) and 470 nm (**D**). The spectra (**A, B**) were normalized to the area of 100 under the spectrum, and the difference spectra (**C, D**) were calculated for the respective excitation spectra. The presented spectra are representative of three separate experiments.

In order to determine the relative contribution of specific complexes to the overall fluorescence pattern in thylakoids isolated from pea and bean leaves, the steady-state fluorescence emission spectra at 77 K were normalized to the same area (100) under the spectrum (Figure 6A, B). The CP core complexes bind Chl *a* only [4,8]. The Chl *a*/*b* ratio in LHCI was estimated to about 3.5 [10], while in LHCII trimers only to about 1.3 [26,27]. This indicates that the fluorescence emission excited at 412 nm originated mainly from the PSI and PSII core complexes and only slightly from antenna complexes. On the other hand the emission from LHCII increases significantly under the excitation at 470 nm and therefore the fluorescence may be related to the abundance of this complex. The spectra of both species exhibited a common maxima at around 682 nm, 695 nm (visible as a shoulder in pea) and 733 nm. However, the ratio between these main bands in pea and bean spectra is different (Figure 6A, B). The thylakoid emission spectrum is highly heterogeneous and might be resolved in some partially overlapping emission bands corresponding to specific CP complexes [10,28]. The best Gaussian fit to fluorescence spectra enable to distinguish five components (Additional file 4: Figure S2). The bands at around 682 originated from both trimers and monomers of LHCII and CP43 intrinsic antenna of PSII [29,30]. The bands at 695 correspond to emission from the PSII core and to CP43 intrinsic antenna [30]. Aggregated forms of LHCII, disconnected from PSII reaction centers show an additional band at 703 nm. Two bands in the far red emission region at 734 nm and 753 nm are related to the core and antenna of PSI respectively. Thus, integrated intensities of the individual bands at 682 and 695 nm correspond to LHCII-PSII, while both of fluorescence bands at 734 and 753 were related to LHCI-PSI complexes.

In the spectra excited at 412 nm (Chl *a*) the PSI-LHCI to PSII-LHCII emission ratio was slightly lower in pea (3.32) than in bean (3.62) thylakoids. Since most of Chl *a* is bound to core complexes, these data indicate a similar proportion between PSI and PSII core complexes in both species. Under the excitation at 470 nm (Chl *b*) the ratio of (F734 + F753) to (F682 + F695) decreased significantly in pea (2.32) but not in bean (3.20). Furthermore, the ratio F681/F695 following excitations at 412 and 470 nm increases from 4.5 to 6.0 in pea, whereas is stable in bean (1.1 to 0.96). Because most of Chl *b* is located in LHCII [10,26,27], it is suggested that higher abundance of LHCII connected with PSII exists in pea than in bean thylakoids. Similar conclusions may be drawn from the pea minus bean differences spectra (Figure 6C, D), where positive band at around 680 nm attributed to the LHCII complexes [28,29] is observed.

The difference pea - minus - bean spectrum (Figure 6C, D) of the normalized fluorescence emission spectra exhibited a negative band around 700 nm attributed to the emission from multiaggregated forms of LHCII disconnected from the PSII reaction centers [31,32], suggesting a larger concentration of these complexes in bean. Furthermore, the emission difference spectrum showed a positive band at around 720 nm and a broad negative band at around 750 nm (Figure 6C, D), corresponding to the core complex and the antennae of PSI [28]. These data indicate distinct differences in the LHCI-PSI structure between the two species; probably due to the presence of a number of Lhca1-3 isoforms (cf. Figure 3).

**Figure 7:**
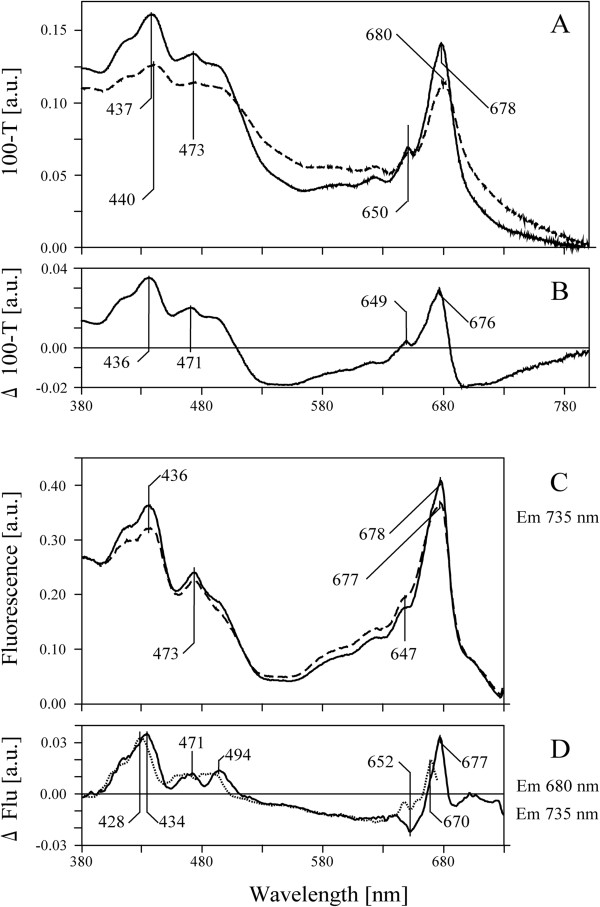
**100-T spectra and fluorescence excitation spectra at 120 K of pea and bean thylakoids.** The 100-T (**A**) and fluorescence excitation centered at 735 nm (**C**). Spectra for pea (solid line) and bean (dashed line) thylakoids (3 μg Chl ml^-1^) were normalized to the area of 100 under the spectrum. Arithmetic difference spectra pea-minus-bean, were calculated for 100-T spectrum (**B**) as well as for fluorescence excitation spectra centered at 680 nm (dotted line) and 735 nm (solid line) (**D**).

Steady-state electron absorption spectra revealed the properties of pigments bound in CP complexes [33]. The analysis of relative contributions of individual bands in one minus transmission spectra (100-T) at 120 K, normalized to the same area (100) under the spectrum, was performed for pea and bean thylakoids (Figure 7). Pea thylakoids spectrum showed maxima at 437 nm (Chl *a*), 473 nm (Chl *b*) as well as at 678 nm (Q_y_ of Chl *a*), whereas the bean thylakoids spectrum revealed red-shifted bands for Chl *a* (440 and 680 nm), but not for Chl *b* (473 and 650 nm) (Figure 7A). The difference absorption spectra of pea thylakoid membranes relative to those of bean thylakoids (Figure 7B), exhibited positive bands at Soret and Q_y_ regions broad negative bands around 530 and 720 nm (Figure 7B).

Normalized excitation spectra presented the relative energy transfer from the absorbing pigments to the emitting Chl species reflecting the state of CP complexes [33]. The spectra at 120 K (Em 735 nm) of pea and bean thylakoids revealed typical excitation bands in the Soret region (400–500 nm) due to light-harvesting by Chl *a* (436 nm), Chl *b* (473 nm), and carotenoid pigments, as well as bands in the red wavelengths solely due to the excitation of Chl *a* (677–678 nm) and Chl *b* (647 nm) (Figure 7C). Difference excitation spectra between respective pea and bean spectra centered at 680 or 735 nm showed significant positive bands at around 430, 470 and 490 nm in the Soret region and at Q_*y*_ bands of Chl *a* (Figure 7D).

The shape of main bands in the absorbance and excitation spectra (Figure 7A, C) indicates that they are not affected by the light scattering effect in the Soret and chlorophyll Q regions [33]. Thus, positive bands in the excitation difference spectra (Figure 7D) suggest a more efficient energy transfer from the antennae complexes to both Chl species emitting at 680 (PSII) and 735 nm (PSI) in pea than in bean thylakoids, probably due to much higher ratio of LHCII to the PSII core (Figure 6). On the other hand, the wide negative bands, present only in the difference absorption spectrum (Figure 7B), are related to light scattering and sieve effects [33-35], probably due to a less regular structure of bean thylakoids (cf. Figures 1 and 2).

### Analysis of PSII functioning based on Chl *a* fluorescence

**Figure 8:**
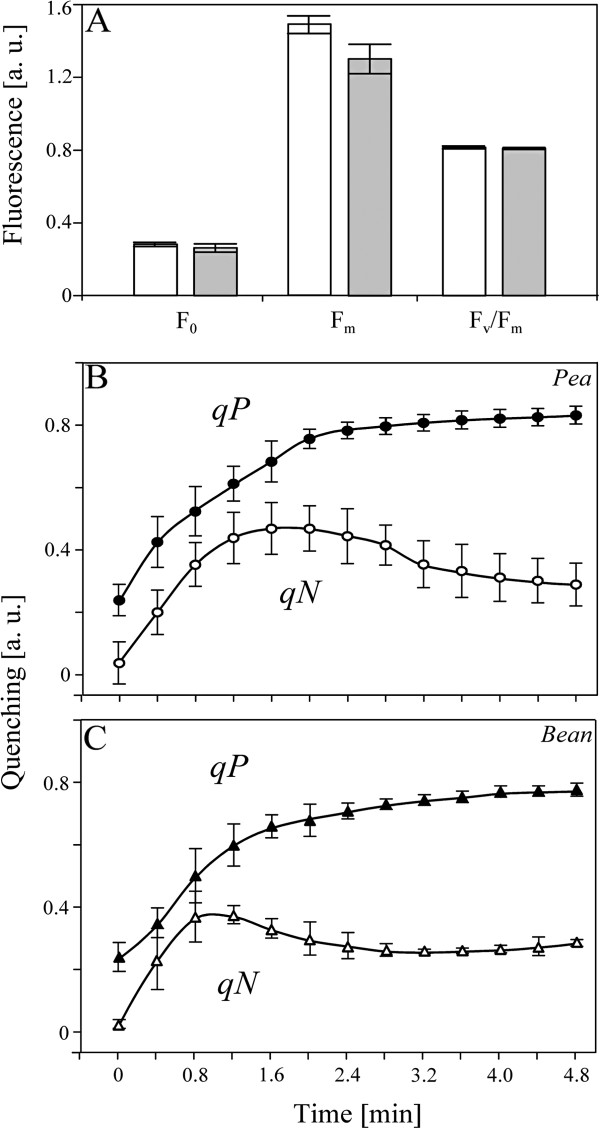
**The PSII functioning of pea and bean measured by PAM fluorescence.** Comparison of F_0_, F_m_ and F_v_/F_m_ parameters for pea (empty bars) and bean (solid bars) (**A**). The rise of quenching parameters in pea (qP - closed circles, qN - empty circles) (**B**) and bean (qP - closed triangles, qN - empty triangles) (**C**). Measurements were performed on dark-adapted pea and bean leaves. The data are mean values ± SD for 3 independent experiments.

The PSII photochemistry *in vivo* was tested by measuring the initial (F_0_) and maximum fluorescence (F_m_) values in dark-adapted pea and bean leaves (Figure 8A). The intensities of Chl *a* fluorescence with open reaction centers (F_0_) were similar in both species, and with closed reaction centers (F_m_) were slightly higher in pea compared with bean leaves. The absolute values of these parameters can be influenced by optical properties of the leaf. Thus were calculated the variable fluorescence values (F_v_), that reflect the PSII photochemistry indicated by the redox state of Q_A_, and the F_v_/F_m_ ratio, that informs about the maximum quantum yield of PSII in the dark-adapted state. The F_v_/F_m_ ratio did not differ significantly between the two species (Figure 8A), indicating the optimization of electron transport and metabolic processes in both plants.

The photochemical quenching coefficient (qP), the PSII efficiency factor, which is non-linearly related to the proportion of open PSII centers (Q_A_ oxidized) at a given light intensity [36], increased similarly in relation to time of illumination (Figure 8B, C). The maximum qP values for pea and bean leaves, reached at saturating light conditions, were estimated at 0.83 and 0.78, respectively.

The non-photochemical quenching parameter (qN), reflecting the apparent rate constant for non-radiative energy dissipation from PSII and its antennae, revealed a slightly different shape in relation to illumination time (Figure 8B, C). The maximum qN values for pea and bean leaves were estimated at 0.47 and 0.37 after five and four consecutive flashes, respectively. Moreover, despite the fact that qN under saturated light conditions was high (about 0.28), the qN declined slower in pea than in bean.

**Figure 9:**
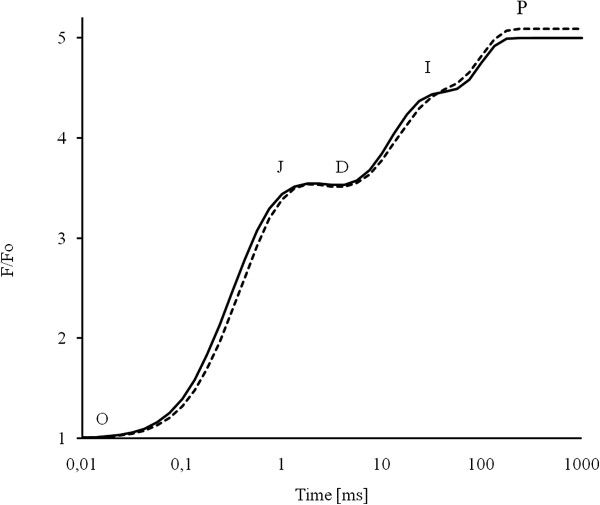
**Chl*a*fluorescence kinetics (OJDIP).** Chl *a* fluorescence induction curves of dark-adapted pea (solid line) and bean (dashed line) leaves.

The polyphasic fluorescence rises in a time domain from 10 μs to 1 s from its origin (O) to its peak (P). The rise has been interpreted, under the strict assumption that photoelectrochemical and photoelectrical events are not involved or ineffective, to reflect the successive filling up of the PSII primary and secondary electron acceptors (Figure 9) [37-41]. Different inflection points of the fluorescence curve have been suggested to correspond to: J –the peak concentrations of Q_A_ Q_B_^-^ and Q_A_^-^ Q_B_^-^, I - the concentration change of Q_A_^-^ Q_B_^2-^ and P – the peak concentrations of Q_A_^-^ Q_B_^2-^ and PQH_2_[42]. This conceptually means, within this concept, that all reaction centers are open at O and all are closed at P, if the light pulses are saturating (then P = F_m_). The basic pattern for fluorescence kinetics holds for all plants. However, the actual pattern depends, again within the concept, on the number of PSII structural and functional features, such as, the state of water oxidation complex or the redox state of the intersystem electron transport chain [43]. Presented description of fluorescence induction curve distinguished a dip phase (D) between the J and I level and is denoted by the so-called OJDIP rise [41]. The Chl *a* fluorescence transients of the dark-adapted leaves of pea and bean are shown on the logarithmic scale from 10 μs up to 1 s (Figure 9). Both curves show the typical OJDIP shape (the O, J, I, D and P steps are marked in the plot). After normalization to F_0_ = 1, the difference between both curves becomes apparent: (i) the J-, (D-,) and P-levels are somewhat higher in the bean leaf, (ii) the dip (D) is a little more pronounced in pea, and (iii) the steepness of D-I phase is different in both leaf types.

**Figure 10:**
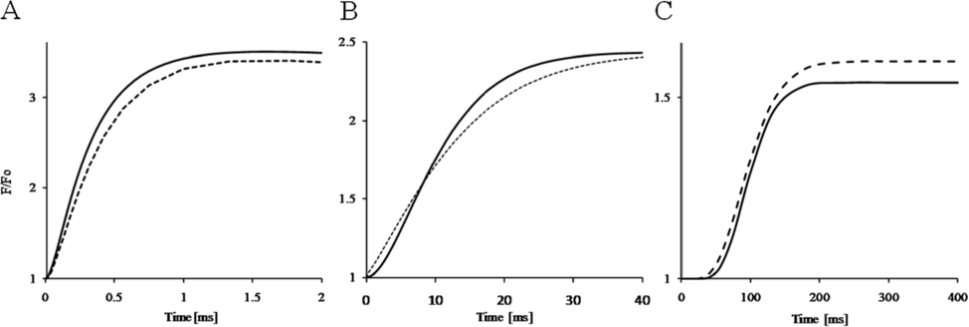
**Analysis of OJDIP kinetics curves.** Time courses of the release of photochemical (**A**), photoelectrochemical (**B**) quenching and of photoelectric fluorescence stimulation (**C**), respectively in dark-adapted pea (solid lines) and bean leaves (dashed lines) calculated with fluorescence induction algorithm. Curves are on a linear time scale.

The alternative interpretation of polyphasic OJDIP curve is that the subsequent kinetic phases are associated with the release of photochemical (OJD) and photoelectrochemical quenching (JDI) and of photoelectrical stimulation (IP), respectively [44]. Application of the fluorescence induction algorithm (FIA) to both experimental curves enables the estimation, illustration and comparison of the respective responses of photochemical (F^PP^), photoelectrochemical (F^PE^) and photoelectrical (F^CET^) processes occurring in these time domains (Figure 10).

Release of the photochemical quenching, F^PP^(t), which is the major contributor of the variable fluorescence F_v_(t) in the O-J-phase (Figure 10A), is associated with about equal maximal variable fluorescence F_v_/F_0_ of 2.4 and 2.5 in bean and pea, respectively. Initial F_v_(t) kinetics are sigmoidal, indicating equal effect of donor side quenching in both species. The rate of F_v_(t) is slightly faster in pea, probably due to a somewhat higher antenna size as compared to bean leaves. The release of photoelectrochemical quenching, F^PE^(t), which is the major contributor of the variable fluorescence Fv(t) in the J-I-phase (Figure 10B), is reflected by a maximal variable fluorescence F_v_/F_0_ ~ 1.4 in both species. The rise kinetics are distinctly different: in bean with nearly exponential kinetics, in pea with a delayed and steeper rise pattern. The photo-electrical stimulation F^CET^(t) causing the final (I-P) phase of the variable fluorescence F_v_(t) with maximal F_v_/F_0_ ~ 0.54 and 0.6 in bean and pea, respectively, occurs with a delay of approximately 50 ms and a steep rise (Figure 10C). The delay and the steepness of F^CET^(t) is somewhat higher in pea. It has been concluded from the FIA-model [44] that an increase in delay and steepness of the fluorescence response during the I-P-phase points to a higher proton buffering capacity at the lumenal side of the CET-driven proton pump. A relation with the size of the granum stack, higher in pea, is suggestive in this respect.

## Discussion

The diversity of chloroplasts membrane system into appressed (stacked) thylakoids and interconnected non-appressed (unstacked) thylakoids is generally described as a consequence of physicochemical interactions between the neighbouring membranes and steric differences between photosystems [5,6,45]. However, complete model of thylakoid membrane network is still under discussion [46,47]. Furthermore, it is not clear what can be the reason of specific arrangements of the thylakoid membranes in various plant species. Our results from CLSM and TEM (Figures 1 and 2) strongly indicate the existence of different arrangements of pea and bean thylakoid membranes. In pea, we showed by CLSM that larger appressed thylakoids are regularly arranged within chloroplasts as uniformly distributed red fluorescent bodies. In contrast, irregularly distributed appressed thylakoid membrane within bean chloroplasts corresponded to smaller and less distinguished fluorescent areas in CLSM images. Recent investigations on selective localization of PSI and PSII fluorescence within chloroplast *in situ* by Hasegawa *et al.*[22] demonstrated that the regions of respective fluorescence areas are separated from each other but a significant amount of PSI emission is merged with PSII fluorescence. This means that in our CLSM images (Figure 1 A, B) LHCI-PSI complex might be localized in both dark and red areas, but the LHCII-PSII and LHCII trimers are exclusively restricted to the glowing red bodies.

3D models of pea chloroplast show a distinct and sharp spatial separation of stacked thylakoids from large stromal spaces (Figure 1C, E, Additional file 1: Video S1). The Spatial division of a stroma and thylakoids areas in bean chloroplast is more complicated; stroma spaces are smaller and very irregular (Figure 1D, F, Additional file 2: Video S2). The Spatial layout of Chl fluorescence forms areas that differ in size and spatial orientation (Figure 1C–F), especially in bean chloroplast, where merging of grana and stroma thylakoids is observed (Figure 2D). These 3D models indicate that the spatial arrangement of the thylakoid membranes within chloroplast is more heterogenous, unordered and more diverse between species than could be concluded from single cross-section observed by TEM [48].

The role of LHCII complexes in formation of grana based on investigation of mutants and shade grown plants have been proposed many years ago [6,16]. The increase of LHCII abundance in thylakoids or its polyaminylation lead to enhancement of the thylakoid stacking due to the increase of the van der Waals attractive force between the adjacent thylakoid membranes [6,49]. The decrease in the content of one or more Lhcbs proteins leads to the increase of the thylakoids disorder due to the increase of electrostatic and hydratation repulsion [6,18]. Furthermore, recent detailed investigations demonstrated that all six Lhcb proteins [18,50] as well as PsbP and PsbQ polypeptides of oxygen evolving complex [17,51] play significant role in the stabilization of LHCII-PSII supercomplexes.

Therefore, to find correlation between the protein composition and the arrangement of pea and bean thylakoids we performed qualitative and quantitative analysis of Lhc and the core proteins as well as the chlorophyll-protein complexes. The immunodetection and MS/MS analysis enabled the identification of seventeen Lhcbs polypeptides in thylakoids of pea and bean (Figure 3A, B). The estimated molecular weights of the Lhcb proteins were similar in pea and bean, but a significant difference in the number and molecular weight of Lhca1-3 isoforms was found (Figure 3C). These observations might indicate that a number of diverse populations of the LHCI-PSI complexes exist both in pea and bean thylakoids. The relative quantitative immunodetection analysis of the selected antennae and the core proteins performed with reference to the total Chl content (Figure 4) provides further evidence that the ratio of the LHCII antennae (Lhcb1) to the PSII core (PsbA) proteins is higher in pea than in bean thylakoids. These data indicate that the diversity in pea and bean chloroplasts ultrastructure (Figures 1 and 2) might result from different quantitative and/or qualitative protein composition (Figures 3 and 4). However, this correlation is probably more complicated due to a complex hierarchical architecture of thylakoids [4].

The basic unit of LHCII-PSII consists of the dimeric form of PSII, two copies of monomeric antennae (Lhcb4, Lhcb5) and two LHCII trimers containing Lhcb1 and Lhcb2 proteins (C_2_S_2_ type) [4,8]. C_2_S_2_M_2_ supercomplex was formed by incorporation of two Lhcb6 and two further trimers [50]. Moreover, up to six LHCII trimers might be associated with PSII dimer (C_2_S_2_M_2_L_2_ type) and the aggregates of LHCII trimers (LHCII)_n_ are detected in grana thylakoids. C_2_S_2_M_2_ supercomplexes are associated in different types of megacomplexes dependent on plant species [4]. Furthermore, C_2_S_2_ together with LHCII is arranged in stacked thylakoids both in random and in ordered arrangements [3,52]. Moreover, the increased of PSII antenna size and membrane numbers in granum as well as formation of semicrystalline LHCII-PSII arrays are observed in low-light grown plants [53]. The LHCI-PSI complexes in unstacked regions of membranes might bound the LHCII trimers, creating new supercomplexes, but its association in megacomplexes is not established yet [54].

Allen and Staehelin [25] have shown that different species revealed different patterns of green bands in mild-denaturing electrophoresis. Moreover, the content of specific bands is related to the abundance of appropriate proteins, as revealed in Arabidopsis mutants [55,56]. We characterized 10 green bands in pea and bean thylakoids. The first five, with high molecular weight, contained polypeptides corresponding to both LHCI-PSI and LHCII-PSII supercomplexes, while the next three bands with lower molecular weights were attributed to LHCII-PSII and LHCII trimers (Figure 5A, B). Mild-denaturing conditions retained the structure of CP supercomplexes and megacomplexes proportionally to accessibility of detergent to particular membrane domains [11,12,25]. Thus, larger amount of green bands related to LHCII-PSII and LHCI-PSI in bean thylakoids (Figure 5) indicates higher density of membranes and stronger interaction between CP complexes. These observations correlate with previous analysis of the infrared spectrum which demonstrates a higher average protein density in bean than in pea thylakoids [57]. Furthermore, the presence of a high molecular green band and a decrease of grana size were observed in *psae1-1* mutant of Arabidopsis due to accumulation of stable LHCI-PSI-LHCII supercomplex [55]. Therefore we suggest that higher abundance of the dense megacomplexes influences the thylakoids structure in bean (Figures 1 and 2).

On the other hand, the abundance of green bands related to LHCII, loosely and strongly associated with PSII, is significantly higher in pea thylakoids (Figure 5A, C). Analysis of 77 K spectra indicate a similar proportion between the PSI and the PSII core complexes in both species, but higher abundance of LHCII in pea thylakoids (Figure 6). Higher ratio of LHCII/PSII core complexes in pea thylakoids is also confirmed by the apoprotein ratio (Figure 4). Furthermore, the difference fluorescence excitation spectra in Soret and chlorophyll Q regions (Figure 7D) indicates a more efficient energy transfer from antennae to the PSII reaction centers in pea than in bean thylakoids. These data suggest that more LHCII trimers are weakly attached to the PSII supercomplex in pea than in bean. Such organization of CP complexes affects the 3D structure of the whole pea chloroplast, where large condensed areas of stacked thylakoid membranes are observed (Figure 1A, C, E, Additional file 1: Video S1).

A smaller abundance of the LHCII trimers (Figure 5A, C), fluorescence of LHCII antenna (Figure 6B) and smaller relative amounts of Lhcb1 to PsbA apoproteins in bean (Figure 4) in comparison to pea thylakoids were found. The 77 K difference spectra of bean thylakoids (Figure 6C, D) indicated significant amounts of multiaggregated forms of LHCII which are probably located in separated domains disconnected from PSII [28,32]. These observations suggest that a smaller number of LHCII trimers per PSII reaction center is present in bean than in pea thylakoids.

In spite of the differences in chloroplast structure (Figures 1 and 2), protein and CP composition (Figures 4,5 and 6) the photosynthetic efficiency represented by qP is similar in both plants. The parameters qP and qN are not directly related: the former is relevant only to the illuminated state of the sample, whereas the latter describes both the light- and dark-adapted states [36]. A slight difference in qN (Figure 8) and the moment in which the maximum value of qN is achieved, may suggest distinct reaction to light in the studied plants. These data suggest that bean plants are better adapted to dark–light changes than pea.

Non-photochemical quenching can generally be subdivided into three components: feedback de-excitation (qE), photoinhibition (qI) and state transition (qT) [58]. In the context of the present study, qE is of interest, since it is the only component connected with changes induced by high-light exposure on a time scale of a few seconds to minutes [59]. Arabidopsis antisense plants lacking most of the Lhcb1 and Lhcb2 proteins exhibited a reduced qE [60]. However the reduction of qE is less pronounced in these plants than was reported for intermittent light-grown plants from pea, bean and barley [61]. Moreover, in the *npq5* mutant of *Chlamydomonas reinhardtii* that lacks specifically the *lhcbm1* gene, qN was drastically reduced [62]. The simplest interpretation of these data is that the generation of maximum qE requires a scattered organization of the PSII antenna including major LHCII. Studies of Mg^2+^ induced stacking of isolated thylakoid membranes of spinach proved that grana stacking is of high importance for the process of overall non-photochemical quenching [63]. Maybe because of that the PSII complexes in the stacked grana membranes posses a larger functional antenna compared with the PSII complexes in the partially and completely unstacked thylakoids [63].

The detailed analysis of polyphasic fluorescence curves (Figure 9) and the comparison of those of the respective underlying processes (Figure 10) suggested a larger functional PSII antenna size [44,64] in pea than in bean thylakoids. Moreover, a different chemical environment, notably a higher number of proton buffering groups, concluded from photoelectrochemical quenching (F^PE^) and photoelectrical stimulation (F^CET^) (Figure 10), suggest higher granum stack in pea chloroplasts [44]. Thus, data obtained by both modulated and transient fluorescence (Figures 8,9 and 10) are in agreement with low temperature spectrophotometry (Figures 6 and 7) and quantitative CP analysis (Figures 4 and 5).

The size and/or the number of LHCII-PSII complexes play a crucial role in the stabilization of 3D grana stacks [65]. This relation is seen as large areas for pea and small areas for bean of Chl fluorescence corresponding to grana stacks (Figure 1A, B). Functional analysis confirmed these structural differences (Figure 8,9 and 10). However, computer models prove that this 3D relationship is more complicated (Figure 1C, E, D, F) (Additional file 1: Video S1 and Additional file 2: Video S2).

It is known that supercomplexes are organized in different megacomplexes [4] and the vertical structure of thylakoids is determined by the LHCII-PSII and LHCII arrangements [4,52,66]. Thus, different distances between the neighbouring membranes in grana stacks, estimated to 21 nm for pea and 16 nm for bean (Figure 2C, F) might be a result of a specific arrangement of large and small LHCII-PSII supercomplexes [4,52,53,66]. On the other hand, it is hard to explain larger dispersion of Chl fluorescence (Figure 1B) and large irregularity in 3D model for bean chloroplast as compared to pea (Figure 1D–F, Additional file 2: Video S2). Probably, a more complicated chloroplast structure might be related to a more complex arrangement of CP complexes (Figures 3 and 5).

Based on spectroscopic analysis and 3D models we reported recently [57], that the differences in the thylakoid arrangement between pea and bean chloroplasts influence the stacking process *in vitro*. In pea chloroplasts the increase of magnesium ion concentration changed the degree of membrane appression from wrinkled continuous surface to distinguished stacked areas and a significant increase of the inter-grana area, whereas in bean chloroplasts less pronounced tendencies towards formation of the appressed regions were observed [57].

The protein-protein and lipid-protein interactions play an important role in the formation of the LHCII-PSII megacomplexes in grana thylakoids and influence protein diffusion coefficient [65,67]. Recently, based on analysis of infrared spectra, we detected lower protein/lipids ratio in pea than in bean thylakoids [57]. Since about 60 % of total-lipids contribute to the boundary lipids [68], the amounts of the bulk lipid phase is higher in pea thylakoids but the average protein density is higher in bean [57]. Thus, the differences in the arrangement of supercomplexes between species may be a consequence of both lipid and protein content stoichiometry. However, the diversity of protein isoforms (Figure 3) may play a role in interactions between CP complexes [4].

The main goal of our research was to find the relationship between the spatial chloroplast structure detected by CLSM *in situ* and the arrangements of the CP complexes within the thylakoid membranes. Our 3D computer models of chloroplasts differ from typical chloroplast depictions [48]. We showed that stacked areas are noticeably irregular with variable thickness, merging with each other and are not always parallel to each other.

## Conclusions

Qualitative and quantitative analysis of chlorophyll-protein complexes as well as spectroscopic investigations suggested a close proportion between PSI and PSII core complexes in pea and bean thylakoids, but higher abundance of LHCII antenna in pea. Moreover, higher accumulation of the aggregated form of LHCII in bean thylakoids and distinct differences in the composition of LHCI-PSI between the two species were found. Such composition of membrane induces formation of large stacked domains in pea and many smaller more heterogeneous regions in bean thylakoids. Structural diversity influenced the PSII photochemistry and caused larger functional PSII antenna size in pea, but did not significantly change its photosynthetic efficiency.

We postulate that the differences in chloroplast structure between species are a consequence of quantitative proportions between the individual supercomplexes, their size and arrangement inside membranes.

## Methods

### Plant materials and growth conditions

Pea (*Pisum sativum* L. *cv*. Demon) and bean (*Phaseolus vulgaris* L. *cv*. Eureka) plants (both from PlantiCo Zielonki, 05–082 Babice Stare, Poland) were grown in 3 liter perlite-containing pots in a climate controlled room (22°C/20°C day/night temperature) at a photosynthetic active radiation (PAR) of 200 μmol photons m^-2^ s^-1^ during a 16-h photoperiod and a relative humidity of 60-70 %. Plants were fertilized with full Knop’s nutrient solution. Fully expanded leaves of 18 and 14 day-old pea and bean, respectively, were harvested 30 minutes after the light had been turned on.

### Preparation of thylakoid membranes and of intact chloroplasts

Thylakoid membranes were isolated by homogenization of pea and bean leaves in a buffered isotonic medium and subsequent osmotic shock as described previously [12]. Intact chloroplasts were isolated in a semi-frozen 20 mM Tricine-NaOH (pH 7.5) buffer containing 330 mM sorbitol, 15 mM NaCl, 4 mM MgCl_2_ and 40 mM ascorbate by gentle homogenization of pea and bean leaves. After filtration intact chloroplasts were centrifuged at 2000 *g* for 3 minutes. Pellet obtained in such a way was very gently resuspended in a small amount of 20 mM HEPES-NaOH (pH 7.0) buffer containing 330 mM sorbitol, 15 mM NaCl, 4 mM MgCl_2_ for further investigation with the help of CLSM. The integrity of at least 80 % of the chloroplasts was determined by ferricyanide reduction before and after the osmotic shock [69]. Chloroplasts with damaged envelope were not a subject of the CLSM analysis. The concentration of chlorophyll and the Chl *a*/Chl *b* ratio were quantified spectrophotometrically after extraction with 80 % (v/v) acetone [70].

### Confocal laser scanning microscopy and 3D reconstruction

Isolated intact chloroplasts (30 μg Chl ml^-1^) were suspended in 20 mM HEPES-NaOH (pH 7.5) containing 330 mM sorbitol, 6 % (v/v) glycerol, 15 mM NaCl, 4 mM MgCl_2_ and 30 μM 3-(3,4-dichlorophenyl)-1,1-dimethylurea. After 10 minutes on ice and in dark incubation the suspension was placed on a poly-L-lysine layer (1 mg ml^-1^) and immobilized on a microscopic glass. Samples were imaged using Zeiss LSM 510 confocal laser scanning fluorescence microscope equipped with a PlanApo 63×, NA 1.4 objective lens. Excitation was performed at 543 nm from a helium-neon laser. Fluorescence emission was collected through a 560 nm long pass filter, while the confocal aperture was set at 106 μm (1 airy unit). Z-series (94–116 optical slices) of 1024 × 1024 pixels and 8 bit images were collected. Images which were not affected by fluorescence quenching due to the light-exposure in microscope were chosen for computer analysis. To improve the signal-to-noise ratio the data stacks were deconvolved using the AutoQuant X2 software (Media Cybernetics Inc. MD, USA). Three-dimensional models of chloroplasts were created by Imaris 6.1.3 software (Bitplane AG, Switzerland).

### Transmission electron microscopy

Samples of pea and bean leaves were prepared for TEM as described previously [12]. Pieces of about 1–4 mm^2^ were cut from the middle part of leaves. The material was fixed in 2.5 % (w/v) glutaraldehyde in 50 mM cacodylate buffer (pH 7.4) for 2 h, washed in the buffer and placed in a 2 % (w/v) osmium tetroxide at 4°C in 50 mM cacodylate buffer (pH 7.4) for about 12 h. The specimens, dehydrated in a graded acetone series, were embedded in a low viscosity epoxy resin and cut on a Leica UCT ultramicrotome. Sections stained with uranyl acetate and lead citrate were examined with a JEM 1400 electron microscope (JEOL Co. Japan). The length of grana and stroma lamellae as well as the number of membranes per granum were estimated in quadrilateral area of 3 million nm^2^ using Digital Micrograph v. 3.6.5 demo software (Gatan Inc. CA, USA) for 20 independent images.

### SDS-PAGE and immunodetection

Thylakoid membrane proteins were separated by SDS-PAGE in 14-20 % (w/v) polyacrylamide resolving gels supplemented with 12-17 % (w/v) sucrose, 0.1 % (w/v) SDS and 0.42 M Tris–HCl (pH 9.2). Acrylamide linear gradient with the increasing cross-linking ratio was performed with the help of a peristaltic pump. Stacking gels of 6 % (w/v) were supplemented with 0.1 % (w/v) SDS and 54 mM Tris–HCl (pH 6.1). Thylakoid membranes were solubilized in a 65 mM Tris–HCl denaturing buffer (pH 9.0) containing 0.29 M sucrose, 0.3 M 2-mercaptoethanol, 2.5 % (w/v) SDS, 0.1 % (w/v) bromophenolblue and incubated for 1 minute in 100°C. Samples containing equal amount of chlorophyll (see Figure legends) were loaded to gel wells and resolved using Hoefer SE 400 electrophoresis cell conducted with a 12.5 mM Tris running buffer (pH 8.3) containing 96 mM glycine and 0.05 % (w/v) SDS.

After separation, proteins were transferred to a PVDF membrane (Immun-Blot^TM^, Bio-Rad CA, USA) using an electrophoresis tank filled with a double concentrated running buffer without SDS supplemented with 10 % (v/v) methanol for 45 min at 100 V constant voltage. Next the membranes were blocked for 3 h in a 20 mM Tris–HCl (pH 7.5) buffer containing 0.5 M NaCl and 5 % (w/v) non-fat dry milk and probed with primary polyclonal antibodies with dilutions suggested by the manufacturer (Agrisera, Sweden) for 3 h. The membranes were washed twice for 5 min in TBST buffer (20 mM Tris–HCl, pH 7.5) containing 0.5 M NaCl and 0.1 % (v/v) Tween 20 and a secondary anti-rabbit IgG antibody conjugated with alkaline phosphatase (Bio-Rad CA, USA) diluted at 1:3000 in TBST was used for immunodetection. After membrane washing the immunodetection signals were visualised using *p*-nitroblue tetrazolium chloride and 5-bromo-4-chloro-3-indolyl phosphate *p*-toluidine salt (Color Development Solution, Bio-Rad CA, USA). For PsbA protein secondary anti-chicken IgG antibody conjugated with horseradish peroxidase (Sigma, USA) at 1:5000 dilution in TBST was used and developed with 3,3′-diaminobenzidine (DAB substrate, Roche, Switzerland).

### Protein identification

After the electrotransfer the PVDF membrane was cut in a vertical plane into two pieces and one of them was subjected to the immunodetection as described above and the other piece was placed in a staining solution containing 0.1 % (w/v) Coomassie Blue R-250, 40 % (v/v) methanol, 1 % (v/v) acetic acid for 1 minute. The membrane was washed in 50 % (v/v) methanol in water to remove the excess of dye. For protein location on a gel, blotted and stained parts of the PVDF membrane were compared. The identification of proteins was verified by MS/MS analysis as described by [71].

### Mild-denaturing “green” electrophoresis

Chlorophyll-protein complexes were analysed by mild-denaturing polyacrylamide gel electrophoresis as described by Allen and Staehelin [25] with some modifications. SDS-depleted 3 % (w/v) stacking and 8 % (w/v) resolving polyacrylamide gels supplemented with 10 % (w/v) sucrose and 50 mM glycine were used. Thylakoids membranes (1.26 mg ml^-1^) were solubilized in 2 mM Tris-maleic acid buffer (pH 7.0) containing 10 % (w/v) sucrose, 0.1 % (w/v) lithium dodecylsulphate, 0.92 % (w/v) or 0.65 % (w/v) n-decyl-β-D-maltopyranoside and n-octyl-β-D-glucopyranoside for pea and bean respectively. The detergent concentration used in our experiments corresponded to the most native composition of CP complexes. When the detergent concentration in the incubation buffer was insufficient, the CP complexes with big molecular weights did not penetrate the stacking gel and remained in wells. However, in the case of too high detergent concentration in the incubation buffer, the CP complexes were degraded (not shown). After 20 min. dark incubation on ice, samples containing 31.5 μg of Chl were loaded to gel wells. The electrophoresis was conducted with a 12.5 mM Tris buffer (pH 8.3) containing 96 mM glycine and 0.1 % (w/v) SDS in MiniProtean3 electrophoresis cell (Bio-Rad CA, USA).

For qualitative analysis the gel bands corresponding to CP complexes previously resolved by the “green” electrophoresis were cut and solubilised in a denaturing buffer, incubated for 10 minutes at 100°C and subjected to SDS-PAGE. Polyacrylamide resolving gels of 15 % (w/v) supplemented with 0.1 % (w/v) SDS, 12 % (w/v) sucrose and 5 % (w/v) polyacrylamide containing 0.1 % SDS stacking gels were used. The samples were loaded into gel wells, 25 μl per well, and electrophoresis was conducted with a running buffer in MiniProtean3 tank. Finally the electrophoresis gels were subjected to the immunodetection procedure as described above.

### Gel and blot imaging and quantitative analysis

The resolved polyacrylamide gels as well as the PVDF membranes following immunodetection were scanned with Microteck ScanMarker 5900 in 48 bit RGB, 1200 dpi, and 8 bit gray scale at 2400 dpi resolution. Relative band intensities were quantified using the Quantity One software (Bio-Rad CA, USA). The proteins’ molecular weights were estimated on the basis of the electrophoretical movement rate in comparison with the protein standards (Sigma, USA).

### Low fluorescence and absorbance measurements

Fluorescence emission spectra at 77 K were recorded using a modified Cary Eclipse (Varian Inc., Australia) fluorescence spectrophotometer where excitation and emission beams were led by optical fibers. Thylakoid samples (10 μg Chl ml^-1^) were placed in a polytetrafluoroethylene cuvette and submerged in liquid nitrogen. Excitation wavelength was set at 412 or 470 nm, excitation and emission slits at 5 nm and scans were taken in the range of 600 to 850 nm.

For comparison of absorbance (300–900 nm) and fluorescence excitation spectra (350–675 and 350–735 nm) the samples (3 μg Chl ml^-1^) were measured in the same temperature 120 K in 10 × 10 mm polymethacrylate cuvettes (Sigma, USA) using a homemade liquid nitrogen cryostat (Institute of Physics, Polish Academy of Sciences, Warsaw). Total optical density of the thylakoid samples did not exceed 0.1 to minimize the inner-filter effects [11]. The temperature was measured directly in a glassy solution by a diode thermometer with the accuracy of 0.5 K. Absorbance and excitation spectra were recorded with the Cary 50 Bio spectrophotometer (Varian Inc., Australia) and the Spex FluoroMax spectrofluorimeter (USA) [57], respectively.

During all measurements the thylakoids were resuspended in 20 mM HEPES-NaOH buffer (pH 7.5) containing 15 mM NaCl, 4 mM MgCl^2^ and 80 % (v/v) glycerol.

### Modulated and transient Chl a fluorescence

Chlorophyll *a* fluorescence was measured using a modulated fluorometer (TEACHING-PAM 200, H. Walz GmbH, Germany). The maximum Chl *a* fluorescence level of 30 min dark-adapted leaves (F_m_) was determined during a saturating 1 s flash of 3500 μmol m^-2^ s^-1^ PAR light. The initial Chl *a* fluorescence level of dark-adapted leaves (F_0_) was sensitized with red light of above 1 μmol m^-2^ s^-1^ PAR. The quantum yield of PSII photochemistry (F_v_/F_m_) was calculated from the ratio of variable (F_v_ = F_m_ - F_0_) to the maximum Chl *a* fluorescence. The photochemical [qP = (F_m_’ – F)/(F_m_’ – F_0_’)] and non-photochemical [qN = (F_m_ – F_m_’)/(F_m_ – F_0_)] quenching coefficients were calculated using the pre-programmed sequence of commands and instrumental settings available with the DA-TEACH software (Protocol No.3 in ‘saturation pulse mode’). In this procedure after measuring F_0_ and F_m_, the actinic light (110 μmol m^-2^ s^-1^) was turned on and the fluorescence was measured. A series of saturation flashes (10 ms duration) were applied at 20 s intervals (the complete run took 5 min 20 s), the new F_m_ value (F_m_’) was determined and qP and qN were calculated.

Chl *a* fast fluorescence induction curves were recorded in 30 min dark-adapted pea and bean leaves with a PEA Fluorimeter (Hansatech Instruments Ltd, UK) as described previously [12]. The curves were analyzed by fluorescence induction algorithm with the advanced curve fitting method (GOSA) [44,72].

## Competing interests

The authors declare that they have no competing interests.

## Authors’ contributions

IR, RM, KG, JK-L, BK, BJS, JHV performed the experiments and WPM, WJV, KG, MG analyzed the data. MG designed the experiments. MG, AM, RM, IR, KG, WJV, JHV, WPM wrote the manuscript. All authors read and approved the final manuscript.

## Supplementary Material

Additional file 1 **Video S1.** Animated images (rotation ± 10° around Y axis) of chlorophyll fluorescence of pea chloroplast and 3D reconstruction presented on Figure 1. Click here for file

Additional file 2 **Video S2. **Animated images (rotation ± 10° around Y axis) of chlorophyll fluorescence of bean chloroplast and 3D reconstruction presented on Figure 1. Click here for file

Additional file 3 ** Figure S1. **Protein analysis of pea and bean thylakoid membranes. Full view on SDS-PAGE resolution of thylakoid membrane proteins visualized by staining with Coomassie Blue R-250. Click here for file

Additional file 4 **Figure S2. **Gaussian deconvolution of fluorescence emission spectra at 77 K of pea and bean thylakoids. Pea 412, Bean 412 - samples excited at 412 nm; Pea 470, Bean 470 - samples excited at 470 nm. Spectra were normalized to the area of 100 under the spectrum and subsequently deconvolved into Gaussian bands. The decomposition of fluorescence spectra was performed by using Grams/AI 8.0 spectroscopy Software (Thermo Electron Corporation, USA) programs with 5 Gaussian subbands. Click here for file
