# Supplementary material for: Correlation between spatial (3D) structure of pea and bean thylakoid membranes and arrangement of chlorophyll-protein complexes
Source: BMC Plant Biol. 2012 May 25;12:72. doi: 10.1186/1471-2229-12-72 (PMC3499227; doi:10.1186/1471-2229-12-72)
Supplement: Additional file 3 — Figure S1. Protein analysis of pea and bean thylakoid membranes. Full view on SDS-PAGE resolution of thylakoid membrane proteins visualized by staining with Coomassie Blue R-250. [file 1471-2229-12-72-S3.pdf]

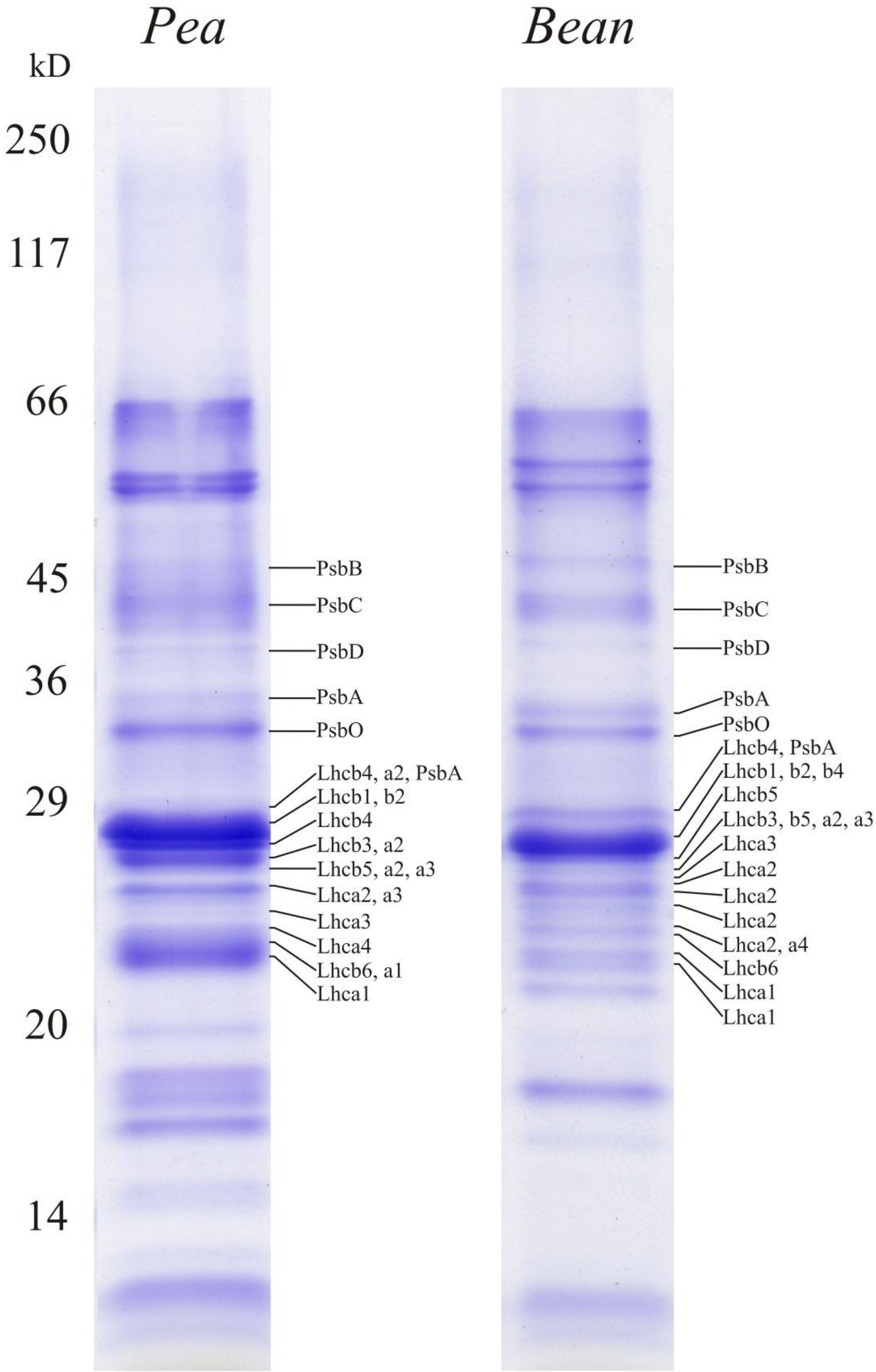

Additional file 3 – Figure 1. Protein analysis of pea and bean thylakoid membranes.
Full view on SDS-PAGE resolution of thylakoid membrane proteins visualized by staining with Coomassie Blue R-250.
