# Supplementary material for: Correlation between spatial (3D) structure of pea and bean thylakoid membranes and arrangement of chlorophyll-protein complexes
Source: BMC Plant Biol. 2012 May 25;12:72. doi: 10.1186/1471-2229-12-72 (PMC3499227; doi:10.1186/1471-2229-12-72)
Supplement: Additional file 4 — Figure S2. Gaussian deconvolution of fluorescence emission spectra at 77 K of pea and bean thylakoids. Pea 412, Bean 412 - samples excited at 412 nm; Pea 470, Bean 470 - samples excited at 470 nm. Spectra were normalized to the area of 100 under the spectrum and subsequently deconvolved into Gaussian bands. The decomposition of fluorescence spectra was performed by using Grams/AI 8.0 spectroscopy Software (Thermo Electron Corporation, USA) programs with 5 Gaussian subbands. [file 1471-2229-12-72-S4.pdf]

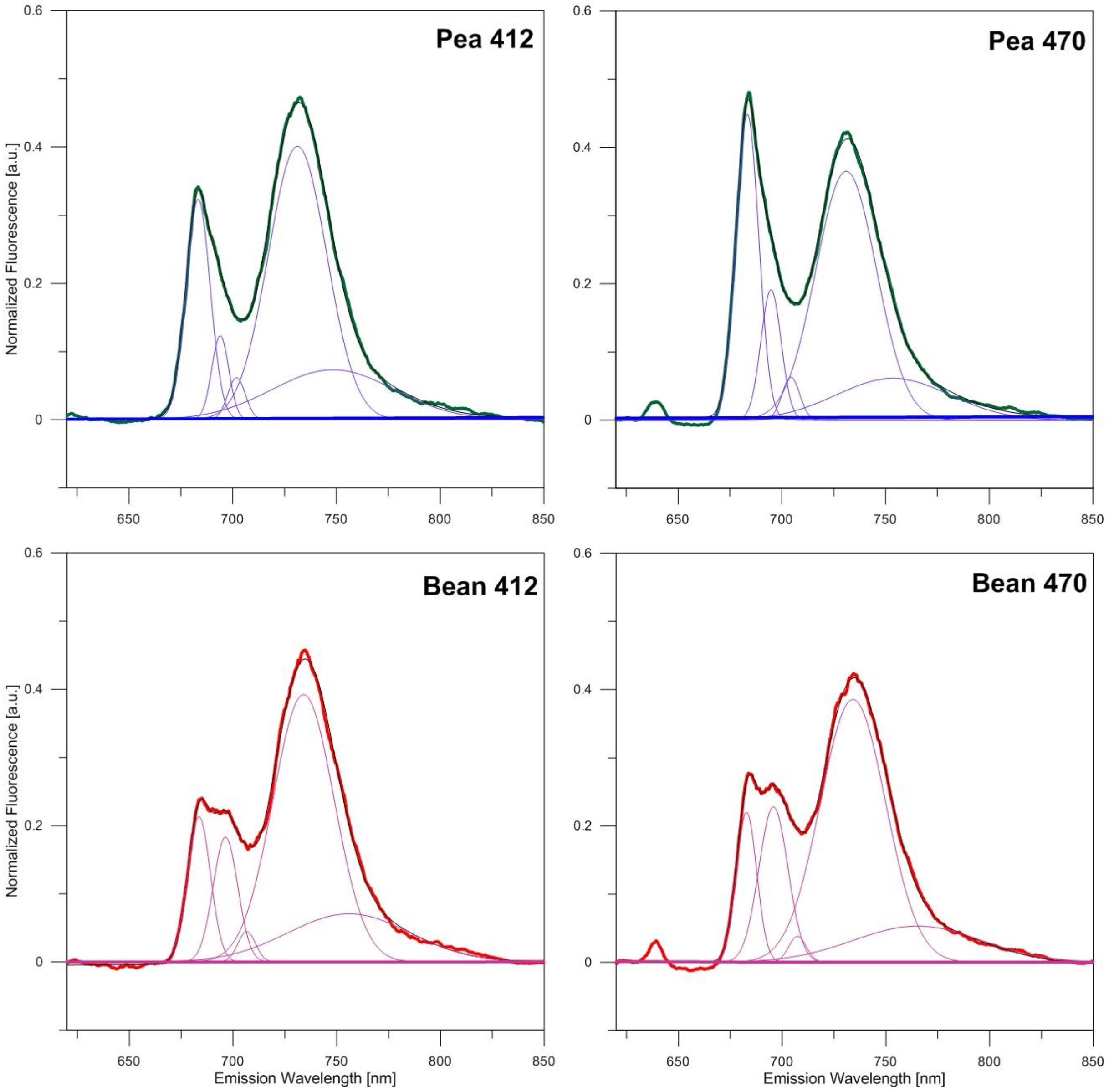

Additional file 4 – Figure 2. Gaussian deconvolution of fluorescence emission spectra at 77 K of pea and bean thylakoids.

Pea 412, Bean 412 - samples excited at 412 nm; Pea 470, Bean 470 - samples excited at 470 nm. Spectra were normalized to the area of 100 under the spectrum and subsequently deconvolved into Gaussian bands. The decomposition of fluorescence spectra was performed by using Grams/AI 8.0 spectroscopy Software (Thermo Electron Corporation, USA) programs with 5 Gaussian subbands
